# Smoking, Corneal Biomechanics, and Glaucoma: Results From Two Large Population-Based Cohorts

**DOI:** 10.1167/iovs.65.1.11

**Published:** 2024-01-03

**Authors:** Kelsey V. Stuart, Kian M. Madjedi, Robert N. Luben, Mahantesh I. Biradar, Siegfried K. Wagner, Alasdair N. Warwick, Zihan Sun, Pirro G. Hysi, Mark J. Simcoe, Paul J. Foster, Anthony P. Khawaja, on behalf of the Modifiable Risk Factors for Glaucoma Collaboration and the UK Biobank Eye and Vision Consortium

**Affiliations:** 1 NIHR Biomedical Research Centre, Moorfields Eye Hospital NHS Foundation Trust and UCL Institute of Ophthalmology, London, United Kingdom; 2 Department of Ophthalmology, University of Calgary, Calgary, Alberta, Canada; 3 MRC Epidemiology Unit, University of Cambridge, Cambridge, United Kingdom; 4 UCL Institute of Cardiovascular Science, University College London, London, United Kingdom; 5 Department of Ophthalmology, St Thomas’ Hospital, King's College London, London, United Kingdom; 6 Department of Twin Research & Genetic Epidemiology, St Thomas’ Hospital, King's College London, London, United Kingdom

**Keywords:** smoking, corneal biomechanics, glaucoma, intraocular pressure, epidemiology, CLSA

## Abstract

**Purpose:**

Smoking may influence measured IOP through an effect on corneal biomechanics, but it is unclear whether this factor translates into an increased risk for glaucoma. This study aimed to examine the association of cigarette smoking with corneal biomechanical properties and glaucoma-related traits, and to probe potential causal effects using Mendelian randomization (MR).

**Methods:**

Cross-sectional analyses within the UK Biobank (UKB) and Canadian Longitudinal Study on Aging (CLSA) cohorts. Multivariable linear and logistic regression models were used to assess associations of smoking (status, intensity, and duration) with corneal hysteresis (CH), corneal resistance factor, IOP, inner retinal thicknesses, and glaucoma. Two-sample MR analyses were performed.

**Results:**

Overall, 68,738 UKB (mean age, 56.7 years; 54.7% women) and 22 845 CLSA (mean age, 62.7 years; 49.1% women) participants were included. Compared with nonsmokers, smokers had a higher CH (UKB, +0.48 mm Hg; CLSA, +0.57 mm Hg; *P* < 0.001) and corneal resistance factor (UKB, +0.47 mm Hg; CLSA, +0.60 mm Hg; *P* < 0.001) with evidence of a dose–response effect in both studies. Differential associations with Goldmann-correlated IOP (UKB, +0.25 mm Hg; CLSA, +0.36 mm Hg; *P* < 0.001) and corneal-compensated IOP (UKB, –0.28 mm Hg; CLSA, –0.32 mm Hg; *P* ≤ 0.001) were observed. Smoking was not associated with inner retinal thicknesses or glaucoma status in either study. MR provided evidence for a causal effect of smoking on corneal biomechanics, especially higher CH.

**Conclusions:**

Cigarette smoking seems to increase corneal biomechanical resistance to deformation, but there was little evidence to support a relationship with glaucoma. This outcome may result in an artefactual association with measured IOP and could account for discordant results with glaucoma in previous epidemiological studies.

Tobacco smoking is a leading cause of global morbidity and mortality and has been implicated as a risk factor for several ocular diseases, including cataract, AMD, and thyroid eye disease.^1^^–^^4^ Evidence for the role of smoking in glaucoma, however, is less clear. Despite multiple population-based studies demonstrating higher IOP in smokers relative to nonsmokers, associations with glaucoma are inconsistent and inconclusive.^5^^–^^8^

Exposure to tobacco smoke has been shown to have detrimental effects on the ocular surface and to induce collagen crosslinking in experimental models.^9^^,^^10^ These physiological and biochemical changes may lead to altered corneal biomechanical properties in habitual smokers, and it has been suggested that this factor could account for an apparent protective effect on keratoconus and other corneal ectasias.^11^^,^^12^

Methods of IOP estimation based on corneal applanation are inherently affected by variability in ocular surface and corneal characteristics, such as tear film adhesion and central corneal thickness.^13^^,^^14^ Any external factor that influences corneal parameters may, therefore, induce an artefactual association with IOP, independent of any true effect on ocular tension. Smoking has been implicated as one such factor that may influence measured IOP through an effect on corneal biomechanical properties, and this finding may explain the lack of a consistent association with glaucoma in epidemiological studies.^15^

To better understand these relationships, we assessed the association of smoking with corneal biomechanical and glaucoma-related parameters in two large population-based cohorts—the UK Biobank (UKB) and the Canadian Longitudinal Study on Aging (CLSA). We additionally conducted two-sample Mendelian randomization (MR) analyses, using results from the GWAS & Sequencing Consortium of Alcohol and Nicotine use (GSCAN) and UKB to probe the potential causal effect of smoking on corneal biomechanics.

## Methods

### UKB

The UKB is a large-scale biomedical database and research resource, derived from a population-based cohort of approximately 500,000 individuals from across the UK.^16^ Participants aged 37 to 73 years were recruited through National Health Service registers and invited to attend one of 22 assessment centers across the UK (2006–2010). After providing electronic informed consent, participants completed a comprehensive touchscreen questionnaire and an array of physical and cognitive measurements. Blood, urine, and saliva specimens were collected and used to generate a wealth of genetic, proteomic, and metabolomic data.^17^ Multiple repeat and supplementary assessments, including an eye and vision substudy (2009–2010), have been conducted on participant subsets to augment the baseline data.^18^ Additional health-related outcomes are available through linkage with nationwide health records and registries. Detailed descriptions, including the overall study protocol and individual test procedures, are available online (https://www.ukbiobank.ac.uk). The UKB was approved by the National Health Service North West Multicentre Research Ethics Committee (06/MRE08/65) and the National Information Governance Board for Health and Social Care. This research was conducted under UKB application number 36741.

### CLSA

The CLSA is a national longitudinal research platform, including approximately 50,000 participants from all 10 Canadian provinces, designed to support a wide variety of aging-related research questions.^19^ Participants aged 45 to 85 years were recruited through random household sampling and invited to join one of two complementary cohorts (2010–2015). After providing written informed consent, a subset of approximately 30,000 (the Comprehensive cohort) completed a detailed in-person home interview and attended 1 of 11 data collection sites, where additional questionnaires, tests, physical measurements, and biological specimens (blood and urine) were collected. Active follow-up occurs every 3 years and record linkage with existing healthcare administrative databases is planned for approximately 90% of the cohort. Further study details, including protocols and test procedures, are available online (https://www.clsa-elcv.ca). Ethical approval for CLSA was granted individually for each data collection site.^19^ This research was conducted under CLSA application number 2109012.

### Smoking-related Exposure Measures

In both the UKB and CLSA, self-reported smoking exposures were derived from a questionnaire administered as a part of the baseline assessment. Participants answered several questions relating to their current and past smoking behaviors, including details of the frequency, intensity, type, duration, and pattern of use. Full details of these assessments, including questionnaire flow and possible responses, are available online for both the UKB (https://biobank.ndph.ox.ac.uk/showcase/) and CLSA (https://www.clsa-elcv.ca/data-collection).

**Figure 1.**
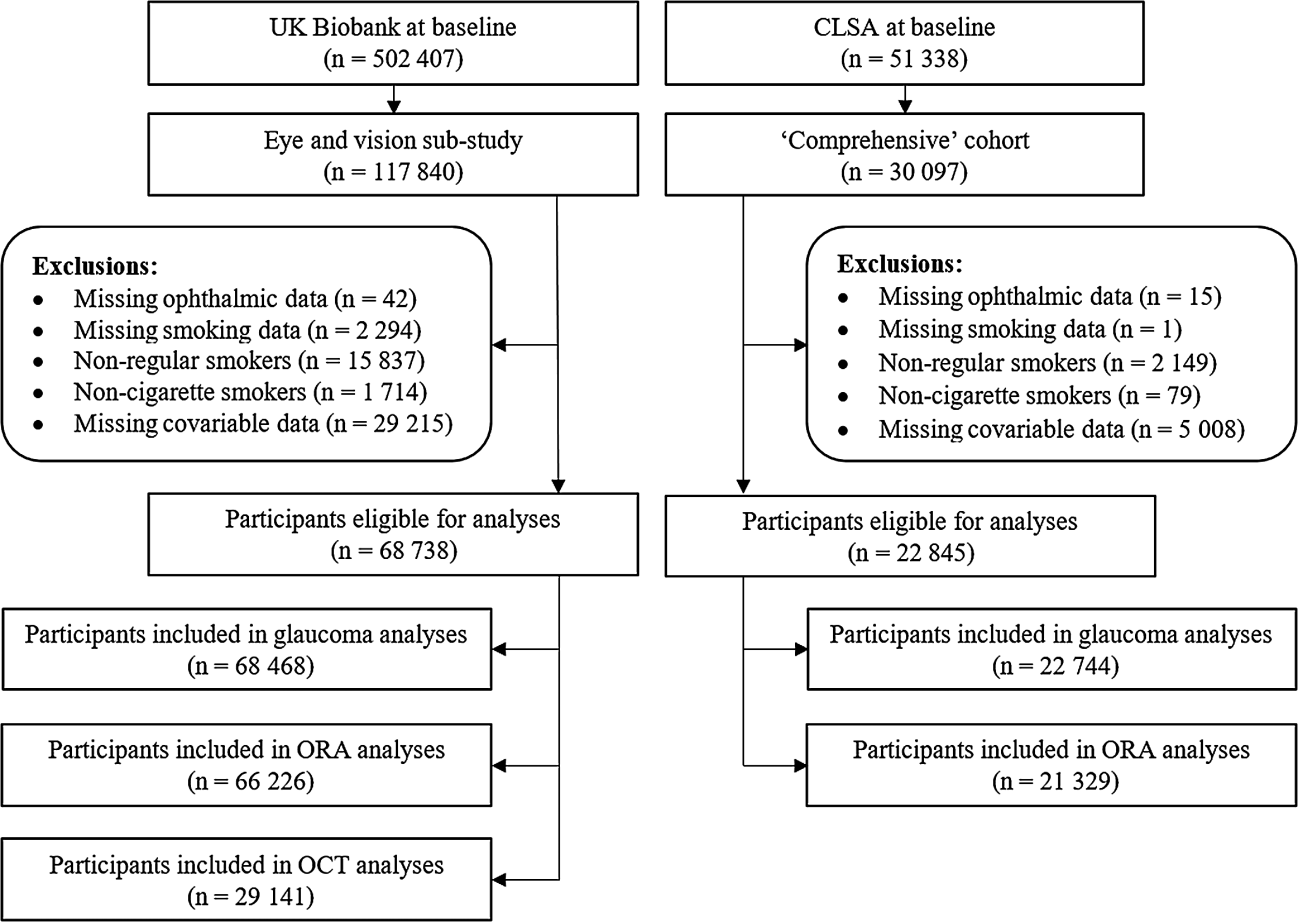
Participant selection and study flow in the UK Biobank and Canadian Longitudinal Study on Aging (CLSA). OCT, optical coherence tomography; ORA, Ocular Response Analyzer.

Smoking status (never, former, current) was defined according to a lifetime exposure to at least 100 cigarettes.^20^ In both studies, quantifiable smoking data were only available for regular (daily or almost daily) cigarette smokers. We, therefore, excluded nonregular and/or noncigarette smokers from the main analyses (Fig. 1), but included these participants in sensitivity analyses of overall smoking status. Smoking intensity (cigarettes/day) was available as a continuous measure in UKB and was categorized (≤5, 6–10, 11–15, 16–20, or >20) for both former and current smokers to align with CLSA data. Smoking duration (years) was categorized separately for former (≤10, 11–20, 21–30, 31–40, of >40) and current (≤30, 31–40, or >40) smokers in both studies.

Pack-years, a quantification of an individual's lifetime exposure to tobacco smoke (1 pack-year is equivalent to 7300 cigarettes), was calculated in UKB as smoking intensity (packs [20 cigarettes]/day) multiplied by smoking duration (years), and was categorized (<10, 10–19, 20–29, 30–39, of ≥40) for both former and current smokers. Passive (secondhand) smoke exposure (hours/week) in never smokers was calculated in UKB as the sum of household and work exposure to other people's tobacco smoke (0, ≤2, 3–10, or >10).

### Corneal Biomechanical and Glaucoma-Related Outcome Measures

**Figure 2.**
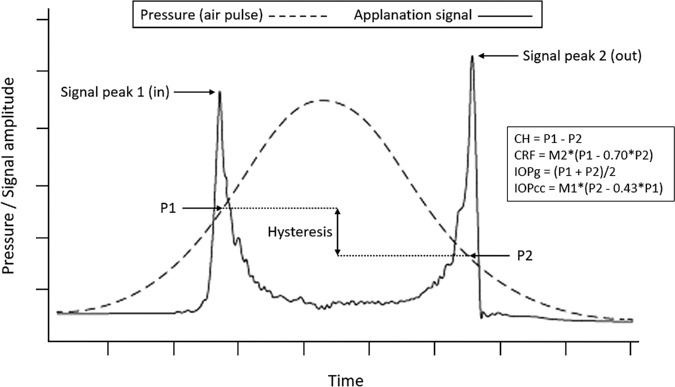
Ocular Response Analyzer pressure profile, illustrating the derivation of the corneal biomechanical (CH, CRF) and IOP (IOPg, IOPcc) parameters used in this study. CH, corneal hysteresis; CRF, corneal resistance factor; IOPG, Goldmann-correlated IOP; IOPcc, corneal-compensated IOP; P1, applanation pressure 1; P2, applanation pressure 2. M1 and M2 are industry calibration constants derived from clinical correlation with Goldmann applanation tonometry.

A subset of approximately 115,000 UKB participants and all approximately 30,000 comprehensive cohort CLSA participants underwent a detailed ophthalmic examination as part of the baseline assessment. The Ocular Response Analyzer (ORA) (Reichert Corp., Philadelphia, PA, USA), used as a part of these assessments, is a noninvasive device that provides measures of both IOP and corneal biomechanics.^21^ A rapid air pulse flattens the cornea, causing an initial inward applanation (P1), followed by an outward applanation event (P2) as the cornea returns to its original shape. An electro-optical system measures the air pressures at these two applanation events and combines them to create four different parameters (Fig. 2). The mean of P1 and P2 is calibrated to provide a measure of IOP closely correlated with Goldmann applanation tonometry (IOPg). A second measure, corneal-compensated IOP (IOPcc), is derived from a linear combination of P1 and P2, and aims to account for corneal biomechanical properties to provide a better reflection of true IOP (Luce D. IOVS 2006;47:ARVO E-Abstract 2266). Corneal hysteresis (CH), the difference between P1 and P2, is a measure of the viscoelastic dampening property of the cornea, and reflects the ability of the cornea to absorb and dissipate energy. Corneal resistance factor (CRF), a complementary measure to IOPcc, is also derived from a linear combination of P1 and P2, and aims to provide a measure of corneal resistance independent of IOP (Luce D. IOVS 2006;47:ARVO E-Abstract 2266). Although the ORA aims to provide independent measures, the biological assumptions and formulae underlying these calculations are based on a small cohort of select individuals, and widespread validity has not been demonstrated.

In both studies, individual-level ORA parameters (CH, CRF, IOPg, and IOPcc) were calculated as the mean of available right and left eye values, and extreme values in the top and bottom 0.5 percentiles of the distribution were excluded. We excluded participants using ocular hypotensive medication (both studies), and those with a history of glaucoma surgery, laser therapy, corneal graft, refractive surgery, or visually significant ocular trauma (UKB only), or recent eye surgery (CLSA only), because these factors may all influence IOP and/or corneal biomechanical properties.

Glaucoma status in the UKB was based on a combination of self-report (glaucoma diagnosis after 30 years of age or previous glaucoma laser/surgical therapy) and *International Classification of Diseases* (ICD) codes for glaucoma (ICD 9th revision, 365.* [excluding 365.0]; ICD 10th revision, H40.* [excluding H40.0] and H42.*) in linked hospital records at any point before, and up to 1 year after, the baseline assessment. To avoid potential misclassification, we excluded controls using ocular hypotensive medication or with an ICD code for glaucoma suspect (ICD 9th revision: 365.0; ICD 10th revision: H40.0). In CLSA, glaucoma status was based on self-report alone.

In an additional subset of approximately 65,000 UKB participants, macular spectral domain optical coherence tomography (OCT) imaging was performed using a Topcon 3D OCT-1000 Mark II system (Topcon Corp., Tokyo, Japan). The image handling, segmentation and quality control protocols have been described previously.^22^ For this study, we assessed associations with two glaucoma-related OCT biomarkers—macular retinal nerve fiber layer (mRNFL) and ganglion cell inner plexiform layer (GCIPL) thickness—using individual-level OCT values from the macula-6 grid averaged across both eyes.^23^^,^^24^ OCT imaging was not performed in CLSA.

### Covariables

To account for potential confounding bias, we considered a range of factors that may be related to both smoking habits and corneal- or glaucoma-related measures. These variables, selected a priori based on previously reported associations,^15^^,^^25^^,^^26^ were ascertained as part of the baseline assessment in both studies, but varied slightly depending on data availability. Both studies collected age (years), sex (women, men), self-reported ethnicity (White, Black, other), body mass index (kg/m^2^), systolic blood pressure (mm Hg), glycated hemoglobin (mM/M), total cholesterol (mM/L),^27^ alcohol intake (g/day),^28^ and assessment season (Summer, Autumn, Winter, Spring). In the UKB only, the following variables were collected: Townsend deprivation index (a measure of material deprivation based on an individual's residential postcode) and spherical equivalent (diopters). In CLSA only, the following variables were collected: highest level of education (less than secondary, secondary/no tertiary, secondary/some tertiary, tertiary) and total household income (C$; <50K, 50–100K, 100–150K, or >150K).

### Statistical Analysis

Baseline participant characteristics were summarized as mean (SD) or median (interquartile range) for continuous variables and frequency (proportion, %) for categorical variables. Normality of continuous data was assessed graphically with histograms and P–P plots. Differences in participant characteristics by cohort were tested with a two-sample *t* test, Wilcoxon rank-sum test, or *z*-test of proportion, as appropriate. To assess the associations of the smoking-related exposures with the various corneal- and glaucoma-related outcomes, we used multivariable linear (for CH, CRF, IOPg, IOPcc, mRNFL, and GCIPL) and logistic (for glaucoma) regression models, with adjustment for all covariables described elsewhere in this article. In the analyses of smoking status, former and current smokers were compared with those who had never smoked. Subsequent analyses of smoking intensity (cigarettes/day) and smoking duration (years) were performed separately in former and current smokers, using those with the lowest exposure as the reference category. Trends across ordinal categories were examined by testing the median value of each group. Statistical tests were two sided and all analyses were performed using Stata (Version 17.0. StataCorp LLC, 2021, College Station, TX, USA).

### MR

**Figure 3.**
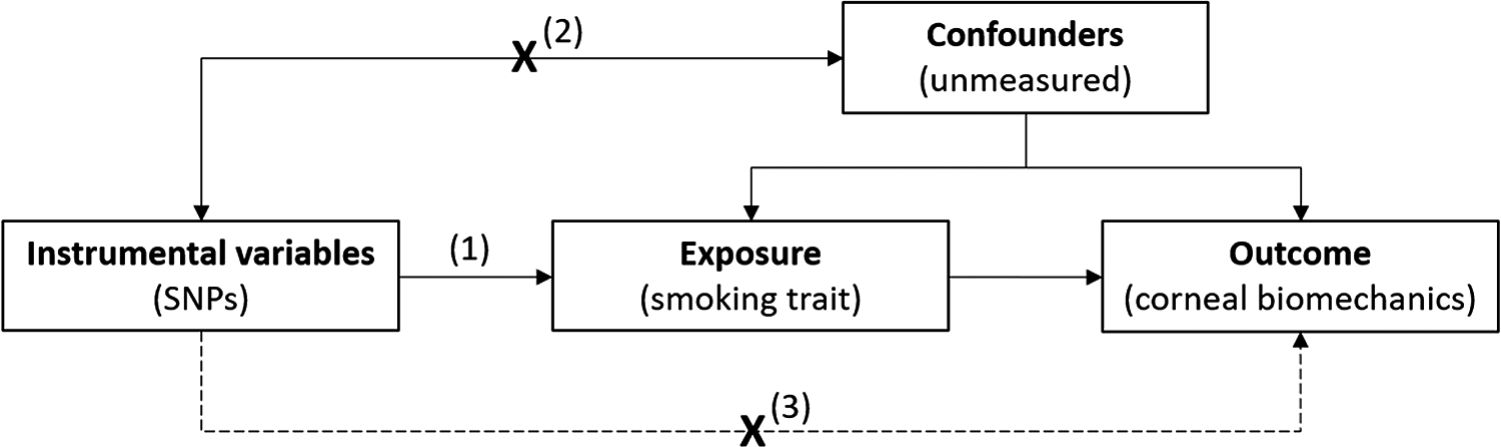
Directed acyclic graph, illustrating the principles and assumptions of the Mendelian randomization framework applied to this study. SNP, single nucleotide polymorphism. Instrumental variable (IV) assumptions: (1) IV is associated with the exposure of interest, (2) IV is not associated with confounders of the exposure–outcome association, (3) IV only affects the outcome via the exposure and not through alternative pathways.

MR is an instrumental variable (IV) technique used to evaluate potentially causal relationships from observational data.^29^ Genetic variants associated with an exposure of interest are used to construct an IV that reflects an individual's lifetime susceptibility to that exposure. The random allocation of genetic variants at conception is analogous to a randomized controlled trial, making MR relatively immune to bias from confounding and reverse causation. Provided that certain assumptions are satisfied, estimates from MR analyses reflect the causal association between a genetically determined risk factor and the development of a particular outcome over the course of a lifetime (Fig. 3).

We performed two-sample MR analyses in participants of European ancestry using results from GSCAN for smoking initiation (a binary phenotype indicating whether an individual had ever smoked regularly; *n* = 249,171) and smoking intensity (cigarettes/day; *n* = 143,210).^30^ To avoid participant overlap, which may bias MR estimates, and owing to data sharing restrictions, we used summary statistics excluding participants from UKB and 23andMe. Corneal biomechanical outcomes were drawn from a recent genome-wide association study for CH (*n* = 106,041) and CRF (*n* = 106,030) in the UKB.^31^ MR analyses for the effect of these traits on glaucoma-related outcomes have been reported elsewhere.^32^ Smoking-related IVs were constructed by selecting all genome-wide significant (*P* < 5 × 10^−8^) single nucleotide polymorphisms (SNPs) and clumping to exclude those with a linkage disequilibrium R^2^ > 0.001 and within 10,000 kb, using the 1000 Genomes Project European reference population.^33^ Effect alleles were harmonized across exposure and outcome datasets and palindromic SNPs with minor allele frequency of more than 0.42 were excluded.

The main MR analyses were performed using a multiplicative random-effects inverse-variance weighted (IVW) approach.^34^ Four alternative MR methods were employed as sensitivity analyses: weighted median, weighted mode, MR-Egger, and MR pleiotropy residual sum and outlier.^35^^–^^38^ We calculated the mean F-statistic as an indicator of instrument strength, and performed relevant tests of heterogeneity, directional pleiotropy, and regression dilution.^37^^,^^39^^,^^40^

### Sensitivity Analyses

We repeated the analyses of smoking status, including all nonregular and noncigarette smokers who were excluded from the main analyses. We additionally considered associations with total lifetime smoking exposure (pack-years) and passive smoke exposure (hours/week) in UKB. To assess the impact of ethnicity on our results, we performed the main analyses of smoking status separately in White and Black participants from both studies. We also repeated MR analyses using the full set of genetic variants (based on genome-wide association study of up to 1.2 million participants) for smoking initiation (378 SNPs) and smoking intensity (55 SNPs) reported in the original GSCAN publication.^30^

## Results

### Participants

Overall, we included 68,738 participants from the UKB and 22,845 participants from CLSA. The study selection process is highlighted in Figure 1 and baseline participant characteristics by cohort are summarized in Table 1. On average, CLSA participants were older (62.7 ± 10.1 years vs. 56.7 ± 8.0 years), more likely to be men (50.9% vs. 45.3%), and of self-reported White ethnicity (94.8% vs. 92.5%) than those from the UKB (*P* < 0.001 for all). CLSA had a higher proportion of former (41.6% vs. 27.1%; *P* < 0.001) and a slightly lower proportion of current (7.3% vs. 8.0%; *P* = 0.001) smokers. The distribution of study participants in each smoking intensity and smoking duration category are available in Table 2, Table 3, and Supplementary Table S1.

**Table 1.** Participant Characteristics by Cohort

Characteristic	UKB	CLSA	*P* Value
Sample size	68,738	22,845	
Age (years)	56.7 ± 8.0	62.7 ± 10.1	<0.001
Sex			<0.001
Women	37,595 (54.7)	11,211 (49.1)	
Men	31,143 (45.3)	11,634 (50.9)	
Ethnicity			
White	63,610 (92.5)	21,646 (94.8)	<0.001
Black	1,833 (2.7)	173 (0.8)	<0.001
Other	3,295 (4.8)	1,026 (4.5)	0.06
Townsend Deprivation Index	−1.1 ± 2.9	—	—
Highest level of education			—
Less than tertiary	—	5,024 (22.0)	
Tertiary	—	17,821 (78.0)	
Total household income (C$)			—
<50,000	—	6,231 (27.3)	
50,000–150,000	—	12,654 (55.4)	
>150,000	—	3,960 (17.3)	
Body mass index (kg/m^2^)	27.3 ± 4.7	28.0 ± 5.3	<0.001
Systolic blood pressure (mm Hg)	137.4 ± 18.3	121.0 ± 16.6	<0.001
Glycated hemoglobin (mmol/mol)	36.1 ± 6.5	38.2 ± 8.2	<0.001
Total cholesterol (mmol/L)	5.7 ± 1.1	5.1 ± 1.1	<0.001
Alcohol intake (g/week), median (IQR)	69.9 (130.4)	40.4 (94.2)	<0.001
Spherical equivalent (diopters)	−0.4 ± 2.7	—	—
Smoking status			
Never smoker	44,636 (64.9)	11,672 (51.1)	<0.001
Former smoker	18,600 (27.1)	9,501 (41.6)	<0.001
Current smoker	5,502 (8.0)	1,672 (7.3)	0.001
Corneal hysteresis (mm Hg)	10.6 ± 1.7	10.1 ± 1.7	<0.001
Corneal resistance factor (mm Hg)	10.7 ± 1.8	10.0 ± 1.8	<0.001
Goldmann-correlated IOP (mm Hg)	15.8 ± 3.3	15.1 ± 3.4	<0.001
Corneal-compensated IOP (mm Hg)	16.0 ± 3.2	16.0 ± 3.4	0.029
mRNFL thickness (µm)	28.9 ± 3.8	—	—
GCIPL thickness (µm)	75.2 ± 5.2	—	—
Glaucoma prevalence	1,128 (1.7)	1,130 (5.0)	<0.001

CLSA, Canadian Longitudinal Study on Aging; GCIPL, ganglion cell inner plexiform layer; IQR, interquartile range; mRNFL, macular retinal nerve fiber layer; UKB, UK Biobank.

All values represent mean ± SD or number (%), unless otherwise specified.

**Table 2.** Association of Smoking Status, Smoking Intensity, and Smoking Duration With Corneal Hysteresis and Corneal Resistance Factor

	Corneal Hysteresis (mm Hg)						Corneal Resistance Factor (mm Hg)					
	UKB			CLSA			UKB			CLSA		
	*n*	β (95% CI)	*P* Value	*n*	β (95% CI)	*P* Value	*n*	β (95% CI)	*P* Value	*n*	β (95% CI)	*P* Value
Smoking status												
Never smokers	42,986	Reference		10,899	Reference		42,980	Reference		10,898	Reference	
Former smokers	17,873	0.10 (0.07 to 0.13)	**<0.001**	8,823	0.10 (0.05 to 0.15)	**<0.001**	17,880	0.12 (0.09 to 0.15)	**<0.001**	8,820	0.11 (0.06 to 0.16)	**<0.001**
Current smokers	5,283	0.48 (0.43 to 0.53)	**<0.001**	1,574	0.57 (0.48 to 0.66)	**<0.001**	5,281	0.47 (0.42 to 0.53)	**<0.001**	1,573	0.60 (0.50 to 0.69)	**<0.001**
Smoking intensity												
Former smokers												
≤5 cigarettes/day	1,032	Reference		1,408	Reference		1,032	Reference		1,407	Reference	
6–10 cigarettes/day	3,712	0.05 (−0.06 to 0.16)	0.41	1,725	0.04 (−0.07 to 0.16)	0.45	3,711	0.02 (−0.10 to 0.14)	0.74	1,723	0.13 (0.01 to 0.25)	**0.033**
11–15 cigarettes/day	3,081	0.09 (−0.03 to 0.20)	0.13	1,487	0.19 (0.07 to 0.31)	**0.002**	3,082	0.02 (−0.10 to 0.15)	0.72	1,487	0.21 (0.08 to 0.34)	**0.001**
16–20 cigarettes/day	6,292	0.12 (0.01 to 0.23)	**0.027**	1,800	0.04 (−0.08 to 0.15)	0.51	6,294	0.06 (−0.06 to 0.18)	0.31	1,800	0.10 (−0.02 to 0.22)	0.11
>20 cigarettes/day	3,650	0.16 (0.04 to 0.27)	**0.007**	2,403	0.22 (0.11 to 0.32)	**<0.001**	3,655	0.11 (−0.01 to 0.24)	0.08	2,403	0.20 (0.08 to 0.31)	**0.001**
*P* (trend)			**<0.001**			**<0.001**			**0.015**			**0.010**
Current smokers												
≤5 cigarettes/day	703	Reference		217	Reference		702	Reference		217	Reference	
6–10 cigarettes/day	1,438	0.18 (0.03 to 0.33)	**0.019**	368	−0.19 (−0.47 to 0.10)	0.20	1,436	0.20 (0.03 to 0.36)	**0.018**	368	−0.10 (−0.41 to 0.21)	0.53
11–15 cigarettes/day	1,293	0.34 (0.19 to 0.50)	**<0.001**	351	−0.06 (−0.35 to 0.22)	0.66	1,293	0.35 (0.18 to 0.52)	**<0.001**	351	−0.06 (−0.37 to 0.25)	0.72
16–20 cigarettes/day	1,197	0.49 (0.33 to 0.65)	**<0.001**	326	0.12 (−0.17 to 0.41)	0.43	1,198	0.43 (0.25 to 0.60)	**<0.001**	324	0.27 (−0.05 to 0.58)	0.10
>20 cigarettes/day	611	0.66 (0.48 to 0.85)	**<0.001**	312	0.58 (0.28 to 0.88)	**<0.001**	611	0.64 (0.44 to 0.85)	**<0.001**	313	0.60 (0.28 to 0.93)	**<0.001**
*P* (trend)			**<0.001**			**<0.001**			**<0.001**			**<0.001**
Smoking duration												
Former smokers												
≤10 years	3,435	Reference		2,915	Reference		3,438	Reference		2,913	Reference	
11–20 years	5,777	−0.01 (−0.08 to 0.06)	0.75	2,607	0.08 (−0.01 to 0.17)	0.07	5,775	0.02 (−0.05 to 0.10)	0.56	2,606	0.07 (−0.02 to 0.16)	0.14
21–30 years	4,409	0.08 (0.00 to 0.15)	**0.039**	1,748	0.16 (0.06 to 0.26)	**0.001**	4,413	0.06 (−0.02 to 0.14)	0.13	1,747	0.15 (0.04 to 0.25)	**0.005**
31–40 years	2,832	0.18 (0.10 to 0.27)	**<0.001**	1,031	0.26 (0.14 to 0.38)	**<0.001**	2,834	0.19 (0.10 to 0.28)	**<0.001**	1,030	0.29 (0.16 to 0.41)	**<0.001**
>40 years	1,267	0.21 (0.10 to 0.32)	**<0.001**	484	0.26 (0.09 to 0.42)	**0.002**	1,267	0.23 (0.11 to 0.34)	**<0.001**	486	0.19 (0.02 to 0.37)	**0.028**
*P* (trend)			**<0.001**			**<0.001**			**<0.001**			**<0.001**
Current smokers												
≤30 years	1,460	Reference		420	Reference		1,458	Reference		420	Reference	
31–40 years	1,834	0.26 (0.12 to 0.39)	**<0.001**	533	0.25 (0.02 to 0.47)	**0.031**	1,833	0.19 (0.05 to 0.34)	**0.010**	533	0.30 (0.06 to 0.55)	**0.013**
>40 years	1,920	0.26 (0.07 to 0.46)	**0.009**	613	0.21 (−0.07 to 0.48)	0.14	1,921	0.19 (−0.02 to 0.41)	0.08	613	0.39 (0.09 to 0.69)	**0.012**
*P* (trend)			**0.006**			0.11			0.06			**0.009**

CI, confidence interval; CLSA, Canadian Longitudinal Study on Aging; UKB, UK Biobank.

Boldface entries indicate *P* values < 0.05.

**Table 3.** Association of Smoking Status, Smoking Intensity, and Smoking Duration With Goldmann-correlated and Corneal-compensated IOP

	Goldmann Correlated IOP (mm Hg)						Corneal Compensated IOP (mm Hg)					
	UKB			CLSA			UKB			CLSA		
	*n*	β (95% CI)	*P* Value	*n*	β (95% CI)	*P* Value	*n*	β (95% CI)	*P* Value	*n*	β (95% CI)	*P* Value
Smoking status												
Never smokers	42,955	Reference		10,894	Reference		42,983	Reference		10,901	Reference	
Former smokers	17,867	0.11 (0.05 to 0.17)	**<0.001**	8,820	0.09 (−0.01 to 0.19)	0.07	17,858	−0.01 (−0.06 to 0.05)	0.85	8,827	−0.02 (−0.12 to 0.08)	0.70
Current smokers	5,283	0.25 (0.15 to 0.34)	**<0.001**	1,569	0.36 (0.18 to 0.55)	**<0.001**	5,282	−0.28 (−0.38 to −0.19)	**<0.001**	1,573	−0.32 (−0.50 to −0.14)	**0.001**
Smoking intensity												
Former smokers												
≤5 cigarettes/day	1,033	Reference		1,405	Reference		1,031	Reference		1,406	Reference	
6–10 cigarettes/day	3,705	−0.04 (−0.26 to 0.19)	0.76	1,723	0.42 (0.18 to 0.66)	**0.001**	3,707	−0.04 (−0.26 to 0.17)	0.69	1,726	0.33 (0.09 to 0.56)	**0.008**
11–15 cigarettes/day	3,080	−0.13 (−0.36 to 0.10)	0.27	1,488	0.21 (−0.04 to 0.46)	0.10	3,082	−0.20 (−0.42 to 0.02)	0.08	1,487	−0.01 (−0.26 to 0.24)	0.93
16–20 cigarettes/day	6,290	−0.07 (−0.29 to 0.14)	0.51	1,803	0.27 (0.03 to 0.51)	**0.029**	6,284	−0.18 (−0.39 to 0.03)	0.09	1,804	0.20 (−0.04 to 0.44)	0.10
>20 cigarettes/day	3,653	−0.05 (−0.28 to 0.18)	0.67	2,401	0.12 (−0.11 to 0.35)	0.29	3,648	−0.20 (−0.42 to 0.03)	0.08	2,404	−0.11 (−0.34 to 0.12)	0.35
*P* (trend)			0.73			0.85			**0.016**			0.06
Current smokers												
≤5 cigarettes/day	702	Reference		216	Reference		703	Reference		216	Reference	
6–10 cigarettes/day	1,437	0.11 (−0.19 to 0.41)	0.48	365	0.08 (−0.52 to 0.67)	0.80	1,436	−0.07 (−0.35 to 0.22)	0.65	368	0.20 (−0.37 to 0.78)	0.48
11–15 cigarettes/day	1,295	0.15 (−0.15 to 0.46)	0.33	350	−0.13 (−0.73 to 0.47)	0.68	1,296	−0.21 (−0.51 to 0.08)	0.15	350	−0.05 (−0.63 to 0.52)	0.86
16–20 cigarettes/day	1,197	−0.07 (−0.38 to 0.24)	0.65	326	0.54 (−0.07 to 1.16)	0.08	1,194	−0.51 (−0.81 to −0.21)	**0.001**	327	0.25 (−0.34 to 0.84)	0.40
>20 cigarettes/day	611	0.16 (−0.21 to 0.53)	0.40	312	0.17 (−0.45 to 0.80)	0.59	612	−0.61 (−0.96 to −0.25)	**0.001**	312	−0.59 (−1.19 to 0.02)	0.06
*P* (trend)			0.93			0.22			**<0.001**			0.07
Smoking duration												
Former smokers												
≤10 years	3,436	Reference		2,913	Reference		3,435	Reference		2,916	Reference	
11–20 years	5,773	0.11 (−0.03 to 0.25)	0.13	2,607	−0.03 (−0.21 to 0.15)	0.72	5,774	0.11 (−0.03 to 0.24)	0.11	2,610	−0.09 (−0.27 to 0.09)	0.32
21–30 years	4,408	−0.01 (−0.15 to 0.14)	0.94	1,745	0.06 (−0.15 to 0.26)	0.54	4,402	−0.09 (−0.23 to 0.05)	0.21	1,746	−0.13 (−0.33 to 0.08)	0.22
31–40 years	2,834	0.08 (−0.08 to 0.25)	0.32	1,033	0.21 (−0.04 to 0.46)	0.10	2,832	−0.12 (−0.28 to 0.04)	0.14	1,033	−0.10 (−0.35 to 0.14)	0.41
>40 years	1,263	0.12 (−0.10 to 0.34)	0.30	485	−0.12 (−0.46 to 0.22)	0.50	1,262	−0.13 (−0.34 to 0.09)	0.24	484	−0.39 (−0.73 to −0.05)	**0.023**
*P* (trend)			0.56			0.42			**0.007**			**0.046**
Current smokers												
≤30 years	1,460	Reference		418	Reference		1,459	Reference		420	Reference	
31–40 years	1,835	−0.14 (−0.40 to 0.13)	0.31	532	0.19 (−0.27 to 0.65)	0.42	1,833	−0.46 (−0.71 to −0.20)	**<0.001**	533	−0.10 (−0.55 to 0.34)	0.65
>40 years	1,918	−0.12 (−0.51 to 0.27)	0.54	611	0.54 (−0.04 to 1.11)	0.07	1,921	−0.43 (−0.80 to −0.06)	**0.023**	612	0.16 (−0.39 to 0.72)	0.57
*P* (trend)			0.51			0.07			**0.015**			0.62

CI, confidence interval; CLSA, Canadian Longitudinal Study on Aging; UKB, UK Biobank.

Boldface entries indicate *P* values < 0.05.

### Associations With Corneal Biomechanics

Compared with never smokers, current smokers had higher CH (UKB, 0.48 mm Hg [95% CI, 0.43–0.53; *P* < 0.001]; CLSA, 0.57 mm Hg [95% CI, 0.48–0.66; *P* < 0.001]) and CRF (UKB, 0.47 mm Hg [95% CI, 0.42–0.53; *P* < 0.001]; CLSA, 0.60 mm Hg [95% CI, 0.50–0.69; *P* < 0.001]). Similar associations, but of a smaller magnitude, were observed in former smokers. In both studies, there was consistent evidence of a dose–response relationship between greater smoking intensity and smoking duration with higher CH and CRF, in both former and current smokers. Full results of these analyses are presented in Table 2.

### Associations With IOP

Compared with never smokers, current smokers had higher IOPg (UKB, 0.25 mm Hg [95% CI, 0.15–0.34; *P* < 0.001]; CLSA, 0.36 mm Hg [95% CI, 0.18–0.55; *P* < 0.001]), but lower IOPcc (UKB, –0.28 mm Hg [95% CI, –0.38 to –0.19; *P* < 0.001]; CLSA, –0.32 mm Hg [95% CI, −0.50 to –0.14; *P* = 0.001]). There was no association of smoking intensity or smoking duration with IOPg in either study. Dose–response associations of greater smoking intensity and smoking duration with lower IOPcc, apparent in the UKB, were not consistently replicated in CLSA. Full results of these analyses are presented in Table 3.

### Associations With Glaucoma

Smoking status was not associated with glaucoma status in either study, or with inner retinal thickness in the UKB. There was also no evidence for a dose–response relationship with either smoking intensity or smoking duration, except for an association between greater smoking duration and a thinner mRNFL in former smokers in the UKB. Full results of these analyses are presented in Supplementary Table S1.

### MR

All genetic variants included in the smoking initiation (10 SNPs) and smoking intensity (9 SNPs) IVs had an *F* statistic of greater than 10 (mean, 36.2 and 100.4, respectively), suggesting sufficient IV strength. Under the IVW method, genetically predicted smoking initiation was associated with higher CH (0.26 mm Hg per SD increase in the IV; 95% CI, 0.13–0.38; *P* < 0.001). This result was supported by both the weighted median and weighted mode approaches. Although the IVW method did not demonstrate a significant association between smoking initiation and CRF, there was evidence for global heterogeneity in this analysis (Cochran's *Q* statistic *P* = 0.025), and alternative approaches able to account for IV heterogeneity (weighted median and MR pleiotropy residual sum and outlier) generated consistent and significant results. Genetically predicted smoking intensity was associated with CH under the weighted median and weighted mode methods, but not with CRF under any approach. Full results of the MR analyses are presented in Table 4 and relevant test statistics in Supplementary Table S2.

**Table 4.** Results of Mendelian Randomization Analyses for Smoking Initiation and Smoking Intensity on Corneal Hysteresis and Corneal Resistance Factor

	Corneal Hysteresis		Corneal Resistance Factor	
MR Method	Estimate (95% CI)	*P* Value	Estimate (95% CI)	*P* Value
Smoking initiation				
IVW	0.26 (0.13 to 0.38)	**<0.001**	0.17 (−0.02 to 0.37)	0.08
Weighted median	0.32 (0.15 to 0.49)	**<0.001**	0.26 (0.05 to 0.47)	**0.016**
Weighted mode	0.36 (0.06 to 0.66)	**0.044**	0.42 (−0.08 to 0.93)	0.13
MR-Egger	−0.56 (−1.45 to 0.33)	0.22	−0.82 (−2.13 to 0.50)	0.22
MR-PRESSO	—	—	0.25 (0.07 to 0.43)	**0.024**
Smoking intensity				
IVW	0.12 (−0.01 to 0.26)	0.07	0.08 (−0.07 to 0.22)	0.29
Weighted median	0.17 (0.02 to 0.32)	**0.022**	0.12 (−0.04 to 0.27)	0.14
Weighted mode	0.22 (0.07 to 0.37)	**0.021**	0.12 (−0.04 to 0.28)	0.17
MR-Egger	0.21 (−0.08 to 0.49)	0.16	0.07 (−0.25 to 0.39)	0.66
MR-PRESSO	—	—	—	—

CI, confidence interval; IVW, inverse variance weighted; IV, instrumental variable; MR, Mendelian randomization; PRESSO, pleiotropy residual sum and outlier; SNP, single nucleotide polymorphism.

Boldface entries indicate *P* values < 0.05.

MR estimates expressed per unit change in the instrumental variable.

No MR-PRESSO estimate is calculated if no significant outliers are detected.

### Sensitivity Analyses

Associations of smoking status were not materially changed when including all nonregular and noncigarette smokers (Supplementary Table S3). In UKB, greater total lifetime smoking exposure (pack-years) was associated with higher CH, higher CRF, and lower IOPcc, in both former and current smokers (*P *trend < 0.001 for all), but not with IOPg. Similar associations with CH, CRF, and IOPcc were also apparent for passive smoke exposure in never smokers (*P *trend < 0.013 for all) (Supplementary Table S4). These analyses also provided evidence for a dose–response association of greater passive smoke exposure with thinner mRNFL and GCIPL in never smokers (Supplementary Table S5). Associations with smoking status were unchanged when restricting analyses to White participants only (Supplementary Table S6). Consistent with the overall results, among Black UKB participants (*n* < 2000), smoking status was associated with higher CH and CRF (in both former and current smokers), but not IOP, inner retinal thickness, or glaucoma status. Estimates derived from the supplementary MR analyses were attenuated but generally consistent with those from the main MR analyses, and provided further evidence to support a causal relationship with CH (Supplementary Table S7).

## Discussion

In this cross-sectional study of two large population-based eye studies, we examined the association of habitual cigarette smoking with corneal biomechanics and glaucoma-related traits. Overall, smoking was consistently associated with a higher CH (greater ability to absorb and dissipate energy) and higher CRF (greater overall resistance) in a dose-dependent manner, with a more pronounced effect in current smokers relative to former smokers. There was also a dose-dependent association of smoking with a lower IOPcc in the UKB, although this finding was not consistently replicated in CLSA. Conversely, smoking status was associated with higher IOPg in both studies, but with no evidence of a dose–response effect. Smoking was not associated with inner retinal thicknesses or glaucoma status in either study. Similar associations were demonstrated when examining total lifetime smoking exposure (in former and current smokers) and passive smoke exposure (in never smokers) in UKB. MR analyses provided evidence for a causal effect of smoking on corneal biomechanics, especially CH.

Acute exposure to tobacco smoke has been shown to have detrimental effects on the ocular surface and tear film function, and certain byproducts of cigarette smoke, including nitrogen oxides, nitrate, and formaldehyde, have been shown to induce collagen crosslinking in experimental models.^9^^,^^10^^,^^41^^,^^42^ This process may lead to permanent corneal changes, with several studies demonstrating altered corneal biomechanical properties in habitual smokers compared with nonsmokers.^11^^,^^43^ This study provides consistent large-scale evidence replicating this association on a population level and, importantly, strong dose-dependent associations and significant MR analyses provide additional evidence to support a causal relationship.

Conversely, cigarette smoke seems to have little short-term effect on IOP, the major modifiable risk factor for glaucoma, or optic nerve head perfusion.^44^ Chronic exposure to harmful compounds found in tobacco smoke has been theorized to influence glaucoma risk though ischemic or oxidative mechanisms, but nicotine has also been hypothesized to be protective through nitric oxide–induced vasodilatory properties.^45^ Although smoking is consistently associated with higher IOP in population-based studies, associations with glaucoma are conflicting and inconclusive.^5^^–^^8^^,^^45^ Because applanation-based methods of IOP measurement may be influenced by structural and functional properties of the cornea, it is possible that smoking-related corneal changes could result in an artefactual association with measured IOP, potentially accounting for the lack of a consistent association with glaucoma.^13^^–^^15^

Consistent with previous reports, current smokers were found to have higher IOPg than never smokers; however, there was no evidence for a dose–response effect, which may have been expected given the strong relationships with CH and CRF. Smoking was also found to be inversely associated with IOPcc in a dose-dependent manner. This differential IOP association has also been reported for several other factors—including ethnicity, height, and diabetes—and suggests that these factors may be particularly related to corneal biomechanical properties.^15^ Similar to diabetes, smoking represents a source of advanced glycosylation end products, which have been shown to induce connective tissue crosslinking and increase tissue rigidity, especially in the presence of glucose.^46^^,^^47^

It is important to acknowledge that measured IOP and corneal biomechanics are inextricably linked, and disentangling these interrelated measures is complex, especially given that all measures are derived from the same device. Although a dose-dependent relationship with a lower IOPcc was observed in this study, and also in previous MR analyses, this finding may be an artefact related to the ORA's correction for corneal biomechanical properties.^32^ Although it remains possible that smoking may have an independent effect on IOP, we found no evidence to support an association between smoking and glaucoma (either adverse or protective) in either cohort, which may have been expected if this were the case.

Interestingly, passive smoke exposure, which has a different chemical composition to that inhaled by active smokers, was found to be adversely associated with inner retinal thickness, especially the GCIPL, in UKB never smokers.^48^ It is possible that the compounds found in passive smoke may have a toxic effect on neural retinal tissue, however, we were unable to replicate these findings in CLSA owing to a lack of OCT data, and given the relatively small participant numbers for these analyses, may represent a chance finding.

In recent years, there has also been significant interest in the role that corneal biomechanics, most notably CH, may play in glaucoma. Individuals with glaucoma have been shown to have a lower CH than healthy controls, and a lower CH is associated with an increased risk of glaucoma progression based on visual fields or structural biomarkers, including in those with apparently well-controlled IOP.^49^ Similar to the limitations discussed elsewhere in this article, the interpretation of these results is complicated by the influence of IOP (inversely related to CH) and topical hypotensive medications on CH measurements, although CH has also been demonstrated to be lower in treatment-naïve patients with normal tension glaucoma compared with healthy subjects with a similar IOP.^49^

The strengths of this study include the large sample size and detailed participant phenotyping available in both the UKB and CLSA, allowing for a simultaneous assessment of associations in two independent cohorts, and across multiple measures of smoking exposure, corneal biomechanics, and glaucoma. This factor enabled us to conduct detailed subgroup and sensitivity analyses, assess for dose–response relationships, and account for important lifestyle and medical factors, such as alcohol consumption and metabolic parameters, which may have biased our results.^27^^,^^50^

Although the main findings of this study were consistent across cohorts, certain results, especially those from analyses involving multiple subgroups and from CLSA in general, were less so. Greater variability in these estimates is likely a result of smaller participant numbers available for these analyses. Although both studies included a detailed smoking questionnaire, this method of exposure ascertainment may be subject to recall and social desirability biases and may not be an accurate reflection of lifetime smoking patterns or behaviors. We were also limited by our method of glaucoma case ascertainment, based on a combination of self-report and electronic medical records, which may be prone to misclassification bias, although this limitation was partly overcome by the availability of quantitative structural OCT biomarkers for a subset of participants. Although the cross-sectional study design limited our ability to assess temporal relationships and make causal inferences, we were able to perform dose–response and MR analyses, which provide alternative approaches to gauge such relationships. Last, our findings in predominantly middle-aged European-descent participants (>90% White ethnicity in both studies) may not be generalizable to other ethnicities or population groups. There are notable regional and ethnic differences in both patterns and methods of tobacco use, and Black individuals in particular have a higher burden of glaucoma and different corneal biomechanical properties relative to White individuals.^51^^–^^53^ This factor may account for the disparate results observed in this study when compared with those conducted in other regions or in more diverse cohorts. Although we did observe suggestive associations between smoking status and corneal biomechanics among Black UKB participants, these analyses were conducted on a relatively small sample (<2000 participants) and it would be important for these results to be replicated in larger cohorts.

Although cigarette smoking is undoubtably detrimental to overall health, this study found little evidence to support an association with glaucoma. Instead, strong associations with CH and CRF, and differential associations with IOPg and IOPcc, suggest a predominant effect on corneal biomechanics, which may induce an artefactual association with measured IOP. Clinicians should be cognizant of this relationship when interpreting applanation-based IOP measures, especially in current smokers. Future research may aim to assess whether similar associations are apparent in e-cigarettes users, especially considering the increasing popularity of this form of smoking in recent years. Recent advances in the development of implantable IOP biosensors may provide further insights into the complex relationship between corneal biomechanics and IOP, by providing a measure of ocular tension independent of potential corneal artefact.^54^

## Supplementary Material

Supplement 1
