# Supplementary material for: Smoking, Corneal Biomechanics, and Glaucoma: Results From Two Large Population-Based Cohorts
Source: Invest Ophthalmol Vis Sci. 2024 Jan 3;65(1):11. doi: 10.1167/iovs.65.1.11 (PMC10768714; doi:10.1167/iovs.65.1.11)
Supplement: Supplement 1 [file iovs-65-1-11_s001.pdf]

Smoking, Corneal Biomechanics, and Glaucoma: Results from Two Large Population-Based Cohorts

SUPPLEMENTARY MATERIAL

Supplementary Table 1. Association of smoking status, smoking intensity, and smoking duration with macular retinal nerve fiber layer thickness, ganglion cell inner plexiform layer thickness, and glaucoma status

Supplementary Table 2. Tests of instrument strength, heterogeneity, directional pleiotropy, and regression dilution for the Mendelian randomization analyses

Supplementary Table 3. Association of smoking status (including all non-regular and/or non-cigarette smokers) with corneal biomechanical and glaucoma-related traits

Supplementary Table 4. Association of lifetime smoking exposure and current passive smoke exposure with corneal hysteresis, corneal resistance factor, Goldmann-correlated intraocular pressure, and corneal-compensated intraocular pressure in the UK Biobank

Supplementary Table 5. Association of lifetime smoking exposure and current passive smoke exposure with macular retinal nerve fiber layer thickness, ganglion cell inner plexiform layer thickness, and glaucoma status in the UK Biobank

Supplementary Table 6. Association of smoking status with corneal biomechanical and glaucoma-related outcomes stratified by self-reported ethnicity

Supplementary Table 7. Results of Mendelian randomization analyses for smoking initiation and smoking intensity on corneal hysteresis and corneal resistance factor using all independent genetic variants reported in the original GSCAN publication as instrumental variables

Supplementary Table 1. Association of smoking status, smoking intensity, and smoking duration with macular retinal nerve fiber layer thickness, ganglion cell inner plexiform layer thickness, and glaucoma status

	**mRNFL thickness (µm)**			**GCIPL thickness (µm)**			**Glaucoma status**					
	**UKB**			**UKB**			**UKB**			**CLSA**		
	N	β (95% CI)	*P*	*N*	β (95% CI)	*P*	*N*	OR (95% CI)	*P*	*N*	OR (95% CI)	*P*
**Smoking status**												
Never smokers	18 601	Reference		18 562	Reference		44 459	Reference		11 625	Reference	
Former smokers	7 998	-0.03 (-0.13, 0.08)	0.61	7 982	-0.01 (-0.14, 0.13)	0.93	18 533	1.10 (0.96, 1.26)	0.17	9 458	1.02 (0.89, 1.16)	0.78
Current smokers	2 227	-0.09 (-0.26, 0.08)	0.28	2 208	0.07 (-0.15, 0.30)	0.53	5 476	1.13 (0.90, 1.43)	0.30	1 661	1.22 (0.94, 1.57)	0.14
**Smoking intensity**												
Former smokers												
≤5 cigarettes/day	446	Reference		445	Reference		1 060	Reference		1 520	Reference	
6–10 cigarettes/day	1 676	0.13 (-0.26, 0.52)	0.51	1 674	0.16 (-0.36, 0.68)	0.54	3 850	1.25 (0.70, 2.24)	0.45	1 833	1.02 (0.75, 1.38)	0.91
11–15 cigarettes/day	1 394	-0.17 (-0.56, 0.23)	0.40	1 389	-0.06 (-0.59, 0.47)	0.82	3 178	1.20 (0.66, 2.18)	0.55	1 583	0.80 (0.57, 1.12)	0.19
16–20 cigarettes/day	2 847	0.01 (-0.36, 0.38)	0.96	2 842	-0.06 (-0.56, 0.44)	0.81	6 534	1.51 (0.87, 2.65)	0.15	1 939	0.86 (0.63, 1.17)	0.33
>20 cigarettes/day	1 592	-0.03 (-0.43, 0.36)	0.87	1 590	-0.07 (-0.60, 0.47)	0.81	3 801	1.42 (0.79, 2.53)	0.24	2 583	1.08 (0.82, 1.44)	0.57
P (trend)			0.49			0.26			0.12			0.73
Current smokers												
≤5 cigarettes/day	326	Reference		326	Reference		731	Reference		229	Reference	
6–10 cigarettes/day	636	0.11 (-0.38, 0.61)	0.65	628	0.35 (-0.32, 1.03)	0.30	1 486	1.34 (0.59, 3.03)	0.49	390	0.83 (0.35, 1.99)	0.68
11–15 cigarettes/day	543	0.07 (-0.44, 0.58)	0.79	538	0.16 (-0.54, 0.86)	0.65	1 336	1.67 (0.74, 3.77)	0.22	372	1.77 (0.81, 3.88)	0.13
16–20 cigarettes/day	490	0.19 (-0.33, 0.71)	0.48	488	0.58 (-0.13, 1.29)	0.11	1 252	1.78 (0.79, 4.04)	0.17	337	1.04 (0.44, 2.48)	0.92
>20 cigarettes/day	219	-0.36 (-1.01, 0.29)	0.27	215	-0.45 (-1.34, 0.43)	0.32	627	0.99 (0.36, 2.73)	0.99	333	1.55 (0.67, 3.58)	0.30
P (trend)			0.61			0.90			0.59			0.23
**Smoking duration**												
Former smokers												
≤10 years	1 539	Reference		1 536	Reference		3 564	Reference		3 095	Reference	
11–20 years	2 638	0.01 (-0.22, 0.24)	0.94	2 624	-0.51 (-0.82, -0.19)	**0.001**	5 987	1.01 (0.74, 1.38)	0.96	2 785	1.43 (1.12, 1.84)	**0.005**
21–30 years	1 989	-0.19 (-0.44, 0.06)	0.13	1 989	-0.29 (-0.63, 0.04)	0.08	4 564	1.00 (0.72, 1.40)	0.99	1 892	1.16 (0.88, 1.54)	0.30
31–40 years	1 259	-0.31 (-0.59, -0.03)	**0.030**	1 261	-0.49 (-0.87, -0.11)	**0.011**	2 949	1.07 (0.75, 1.53)	0.69	1 111	1.33 (0.98, 1.82)	0.07
>40 years	514	-0.32 (-0.70, 0.06)	0.10	513	-0.35 (-0.86, 0.16)	0.18	1 311	1.07 (0.70, 1.62)	0.76	533	1.32 (0.91, 1.91)	0.14
P (trend)			**0.004**			0.12			0.66			0.20
Current smokers												
≤30 years	649	Reference		645	Reference		1 492	Reference		444	Reference	
31–40 years	744	0.15 (-0.30, 0.60)	0.51	736	0.19 (-0.42, 0.80)	0.54	1 899	1.35 (0.65, 2.81)	0.43	556	1.35 (0.67, 2.71)	0.40
>40 years	806	0.32 (-0.32, 0.97)	0.33	799	-0.25 (-1.14, 0.63)	0.58	2 013	1.21 (0.47, 3.09)	0.69	652	0.88 (0.40, 1.92)	0.75
P (trend)			0.33			0.65			0.77			0.68

mRNFL, macular retinal nerve fiber layer; GCIPL, ganglion cell inner plexiform layer; UKB, UK Biobank; CLSA, Canadian Longitudinal Study on Aging; N, sample size; β, beta coefficient; CI, confidence interval; OR, odds ratio.

Supplementary Table 2. Tests of instrument strength, heterogeneity, directional pleiotropy, and regression dilution for the Mendelian randomization analyses

	Corneal hysteresis		Corneal resistance factor	
	Estimate	*P*	Estimate	*P*
Smoking initiation IV (10 SNPs)				
**IVW**				
Mean F statistic	36.2	–	36.2	–
Cochran's Q statistic	8.0 (9)	0.54	19.1 (9)	0.025
I^2 statistic	0.0%	–	52.8%	–
**MR-Egger**				
Rucker's Q' statistic	4.7 (8)	0.79	14.9 (8)	0.06
I^2_{GX} statistic	0.0%	–	0.0%	–
Intercept	0.03	0.07	0.04	0.14
**MR-PRESSO**				
Global test	–	0.57	–	0.030
Number of outliers	0	–	1	–
Distortion test	–	–	-30.2%	0.46
Smoking intensity IV (9 SNPs)				
**IVW**				
Mean F statistic	100.4	–	100.4	–
Cochran's Q statistic	10.0 (8)	0.27	10.1 (8)	0.26
I^2 statistic	19.9%	–	20.9%	–
**MR-Egger**				
Rucker's Q' statistic	9.4 (7)	0.23	10.1 (7)	0.18
I^2_{GX} statistic	96.6%	–	96.6%	–
Intercept	0.00	0.50		
**MR-PRESSO**				
Global test	–	0.29	–	0.36
Number of outliers	0	–	0	–
Distortion test	–	–	–	–

IV, instrumental variable; SNP, single nucleotide polymorphism; IVW, inverse variance weighted; MR, Mendelian randomization; MR-PRESSO, Mendelian randomization residual sum and outlier.

Supplementary Table 3. Association of smoking status (including all non-regular and/or non-cigarette smokers) with corneal biomechanical and glaucoma-related traits

**Trait (study)**	**Never smokers**			**Former smokers**			**Current smokers**		
	N	β / OR* (95% CI)	*P*	*N*	β / OR* (95% CI)	*P*	*N*	β / OR* (95% CI)	*P*
Corneal hysteresis (UKB)	42 986	Reference		27 944	0.06 (0.04, 0.09)	**<0.001**	7 901	0.38 (0.34, 0.42)	**<0.001**
Corneal hysteresis (CLSA)	10 913	Reference		10 036	0.09 (0.04, 0.13)	**<0.001**	2 077	0.51 (0.43, 0.58)	**<0.001**
Corneal resistance factor (UKB)	42 980	Reference		27 948	0.08 (0.05, 0.10)	**<0.001**	7 900	0.39 (0.34, 0.43)	**<0.001**
Corneal resistance factor (CLSA)	10 909	Reference		10 033	0.10 (0.05, 0.15)	**<0.001**	2 076	0.55 (0.47, 0.64)	**<0.001**
Goldmann-correlated IOP (UKB)	42 955	Reference		27 928	0.07 (0.02, 0.12)	**0.006**	7 898	0.21 (0.13, 0.29)	**<0.001**
Goldmann-correlated IOP (CLSA)	10 903	Reference		10 035	0.07 (-0.02, 0.17)	0.12	2 070	0.37 (0.21, 0.53)	**<0.001**
Corneal-compensated IOP (UKB)	42 983	Reference		27 931	0.00 (-0.05, 0.05)	0.94	7 900	-0.22 (-0.30, -0.15)	**<0.001**
Corneal-compensated IOP (CLSA)	10 914	Reference		10 040	-0.02 (-0.11, 0.07)	0.70	2 077	-0.24 (-0.40, -0.08)	**0.003**
Macular retinal nerve fiber layer thickness (UKB)	18 601	Reference		12 505	0.04 (-0.04, 0.13)	0.32	3 417	-0.01 (-0.15, 0.13)	0.91
Ganglion cell-inner plexiform layer thickness (UKB)	18 562	Reference		12 471	0.06 (-0.06, 0.17)	0.35	3 396	0.18 (-0.01, 0.37)	0.06
Glaucoma status (UKB)	44 459	Reference		28 916	1.03* (0.92, 1.17)	0.59	8 177	0.98* (0.79, 1.20)	0.82
Glaucoma status (CLSA)	11 621	Reference		10 754	1.02* (0.90, 1.16)	0.74	2 188	1.20* (0.95, 1.51)	0.12

* Indicates odds ratio. UKB, UK Biobank; CLSA, Canadian Longitudinal Study on Aging; *N*, sample size; β, beta coefficient; OR, odds ratio; CI, confidence interval.

Supplementary Table 4. Association of lifetime smoking exposure and current passive smoke exposure with corneal hysteresis, corneal resistance factor, Goldmann-correlated intraocular pressure, and corneal-compensated intraocular pressure in the UK Biobank

	**Corneal hysteresis**			**Corneal resistance factor**			**Goldmann-correlated IOP**			**Corneal-compensated IOP**		
	N	β (95% CI)	*P*	*N*	β (95% CI)	*P*	*N*	OR (95% CI)	*P*	*N*	OR (95% CI)	*P*
**Lifetime smoking exposure**												
Former smokers												
≤10 pack years	5 323	Reference		5 325	Reference		5 323	Reference		5 324	Reference	
11–20 pack years	5 056	0.04 (-0.02, 0.10)	0.19	5 055	0.04 (-0.03, 0.11)	0.23	5 052	0.04 (-0.09, 0.17)	0.51	5 051	-0.02 (-0.14, 0.11)	0.80
21–30 pack years	3 138	0.13 (0.06, 0.20)	**<0.001**	3 141	0.11 (0.03, 0.19)	**0.008**	3 136	0.01 (-0.14, 0.16)	0.92	3 132	-0.15 (-0.30, -0.01)	**0.035**
31–40 pack years	1 882	0.18 (0.09, 0.27)	**<0.001**	1 883	0.16 (0.06, 0.25)	**0.001**	1 883	0.03 (-0.15, 0.21)	0.75	1 881	-0.18 (-0.36, -0.01)	**0.034**
>40 pack years	1 960	0.28 (0.20, 0.37)	**<0.001**	1 962	0.26 (0.17, 0.36)	**<0.001**	1 960	0.04 (-0.14, 0.22)	0.69	1 957	-0.27 (-0.45, -0.10)	**0.002**
P (trend)			**<0.001**			**<0.001**			0.77			**<0.001**
Current smokers												
≤10 pack years	828	Reference		826	Reference		827	Reference		827	Reference	
11–20 pack years	1 287	0.26 (0.11, 0.41)	**0.001**	1 286	0.28 (0.12, 0.45)	**0.001**	1 285	0.14 (-0.16, 0.42)	0.37	1 285	-0.15 (-0.43, 0.13)	0.28
21–30 pack years	1 205	0.28 (0.13, 0.43)	**<0.001**	1 205	0.34 (0.18, 0.51)	**<0.001**	1 209	0.24 (-0.06, 0.54)	0.11	1 209	-0.08 (-0.36, 0.21)	0.59
31–40 pack years	905	0.59 (0.43, 0.76)	**<0.001**	905	0.51 (0.33, 0.69)	**<0.001**	903	-0.02 (-0.34, 0.31)	0.92	902	-0.57 (-0.88, -0.26)	**<0.001**
>40 pack years	954	0.72 (0.55, 0.89)	**<0.001**	955	0.69 (0.50, 0.87)	**<0.001**	954	0.12 (-0.22, 0.45)	0.49	955	-0.68 (-1.00, -0.36)	**<0.001**
P (trend)			**<0.001**			**<0.001**			0.87			**<0.001**
**Passive smoke exposure**												
Never smokers												
0 hours/week	40 082	Reference		40 076	Reference		40 048	Reference		40 073	Reference	
≤2 hours/week	834	-0.04 (-0.15, 0.07)	0.49	834	-0.10 (-0.22, 0.02)	0.11	834	-0.17 (-0.39, 0.05)	0.13	836	-0.08 (-0.29, 0.13)	0.47
3–10 hours/week	527	0.11 (-0.02, 0.25)	0.11	527	0.11 (-0.04, 0.26)	0.16	527	0.03 (-0.25, 0.31)	0.82	527	-0.07 (-0.33, 0.20)	0.63
>10 hours/week	563	0.29 (0.16, 0.43)	**<0.001**	563	0.21 (0.07, 0.36)	**0.004**	563	-0.07 (-0.34, 0.20)	0.63	564	-0.41 (-0.67, -0.15)	**0.002**
P (trend)			**<0.001**			**0.013**			0.46			**0.003**

IOP, intraocular pressure; *N*, sample size; β, beta coefficient; CI, confidence interval; OR, odds ratio.

Supplementary Table 5. Association of lifetime smoking exposure and current passive smoke exposure with macular retinal nerve fiber layer thickness, ganglion cell inner plexiform layer thickness, and glaucoma status in the UK Biobank

	**mRNFL thickness**			**GCIPL thickness**			**Glaucoma status**		
	N	β (95% CI)	*P*	*N*	β (95% CI)	*P*	*N*	OR (95% CI)	*P*
**Lifetime smoking exposure**									
Former smokers									
≤10 pack years	2 410	Reference		2 401	Reference		5 505	Reference	
11–20 pack years	2 315	-0.08 (-0.29, 0.13)	0.47	2 314	-0.28 (-0.56, 0.00)	0.05	5 245	1.21 (0.90, 1.62)	0.22
21–30 pack years	1 412	-0.24 (-0.49, 0.00)	0.05	1 415	-0.24 (-0.57, 0.09)	0.15	3 271	1.53 (1.12, 2.09)	**0.008**
31–40 pack years	831	-0.41 (-0.71, -0.12)	**0.007**	827	-0.33 (-0.73, 0.07)	0.10	1 946	1.16 (0.79, 1.70)	0.45
>40 pack years	819	-0.29 (-0.60, 0.02)	0.07	816	-0.34 (-0.76, 0.07)	0.11	2 037	1.27 (0.88, 1.82)	0.20
P (trend)			**0.004**			0.07			0.19
Current smokers									
≤10 pack years	376	Reference		374	Reference		857	Reference	
11–20 pack years	584	-0.16 (-0.64, 0.32)	0.52	582	0.00 (-0.66, 0.66)	0.99	1 322	1.66 (0.64, 4.30)	0.36
21–30 pack years	491	0.27 (-0.24, 0.78)	0.29	484	0.02 (-0.68, 0.71)	0.96	1 256	2.77 (1.12, 6.86)	**0.027**
31–40 pack years	365	-0.24 (-0.79, 0.32)	0.40	361	-0.15 (-0.91, 0.61)	0.70	935	2.31 (0.89, 6.01)	0.09
>40 pack years	372	0.11 (-0.47, 0.769	0.72	368	0.10 (-0.69, 0.89)	0.81	996	1.71 (0.64, 4.56)	0.29
P (trend)			0.73			0.97			0.38
**Passive smoke exposure**									
Never smokers									
0 hours/week	17 379	Reference		17 347	Reference		41 439	Reference	
≤2 hours/week	370	-0.35 (-0.74, 0.04)	0.08	365	-0.20 (-0.71, 0.31)	0.44	863	0.87 (0.47, 1.59)	0.65
3–10 hours/week	224	-0.07 (-0.56, 0.43)	0.80	223	-0.32 (-0.97, 0.34)	0.34	551	0.68 (0.30, 1.54)	0.36
>10 hours/week	230	-0.42 (-0.91, 0.06)	0.09	231	-0.93 (-1.57, -0.29)	**0.004**	582	0.57 (0.23, 1.54)	0.22
P (trend)			**0.043**			**0.003**			0.11

mRNFL, macular retinal nerve fiber layer; GCIPL, ganglion cell inner plexiform layer; *N*, sample size; β, beta coefficient; CI, confidence interval; OR, odds ratio.

Supplementary Table 6. Association of smoking status with corneal biomechanical and glaucoma-related outcomes stratified by self-reported ethnicity

**Trait (self-reported ethnicity)**	**Never smokers**			**Former smokers**			**Current smokers**		
	N	β / OR* (95% CI)	*P*	*N*	β / OR* (95% CI)	*P*	*N*	β / OR* (95% CI)	*P*
UK Biobank									
Corneal hysteresis (White)	39 210	Reference		17 208	0.10 (0.07, 0.13)	**<0.001**	4 795	0.50 (0.45, 0.55)	**<0.001**
Corneal hysteresis (Black)	1 363	Reference		205	0.34 (0.10, 0.58)	**0.006**	176	0.39 (0.13, 0.65)	**0.004**
Corneal resistance factor (White)	39 210	Reference		17 214	0.12 (0.09, 0.15)	**<0.001**	4 793	0.49 (0.44, 0.55)	**<0.001**
Corneal resistance factor (Black)	1 359	Reference		205	0.29 (0.03, 0.54)	**0.028**	176	0.49 (0.21, 0.77)	**0.001**
Corneal-compensated IOP (White)	39 208	Reference		17 193	-0.01 (-0.07, 0.05)	0.82	4 794	-0.31 (-0.41, -0.22)	**<0.001**
Corneal-compensated IOP (Black)	1 360	Reference		205	-0.20 (-0.71, 0.30)	0.43	176	-0.01 (-0.56, 0.54)	0.98
Goldmann-correlated IOP (White)	39 186	Reference		17 201	0.11 (0.05, 0.17)	**<0.001**	4 797	0.24 (0.13, 0.34)	**<0.001**
Goldmann-correlated IOP (Black)	1 358	Reference		205	0.14 (-0.37, 0.64)	0.60	176	0.50 (-0.05, 1.05)	0.08
Macular retinal nerve fiber layer thickness (White)	17 152	Reference		7 719	-0.05 (-0.16, 0.05)	0.35	2 042	-0.11 (-0.29, 0.06)	0.21
Macular retinal nerve fiber layer thickness (Black)	511	Reference		81	0.15 (-0.71, 1.00)	0.74	647	-0.15 (-1.17, 0.87)	0.78
Ganglion cell-inner plexiform layer thickness (White)	17 117	Reference		7 705	-0.05 (-0.19, 0.09)	0.47	2 021	-0.02 (-0.26, 0.21)	0.85
Ganglion cell-inner plexiform layer thickness (Black)	508	Reference		80	0.58 (-0.64, 1.80)	0.35	644	1.18 (-0.25, 2.61)	0.11
Glaucoma status (White)	40 566	Reference		17 844	1.09* (0.95, 1.25)	0.22	4 966	1.14* (0.89, 1.46)	0.31
Glaucoma status (Black)	1 414	Reference		211	1.31* (0.57, 3.00)	0.52	191	1.04* (0.37, 2.92)	0.93
Canadian Longitudinal Study on Aging									
Corneal hysteresis (White)	9 799	Reference		8 708	0.10 (0.05, 0.15)	**<0.001**	1 689	0.53 (0.44, 0.61)	**<0.001**
Corneal hysteresis (Black)	108	Reference		35	0.17 (-0.57, 0.91)	0.65	12	-0.08 (-1.18, 1.02)	0.89
Corneal resistance factor (White)	9 797	Reference		8 708	0.10 (0.05, 0.15)	**<0.001**	1 690	0.56 (0.47, 0.66)	**<0.001**
Corneal resistance factor (Black)	108	Reference		35	0.20 (-0.53, 0.94)	0.58	12	0.28 (-0.81, 1.37)	0.61
Corneal-compensated IOP (White)	9 798	Reference		8 712	-0.06 (-0.15, 0.04)	0.26	1 688	-0.28 (-0.45, -0.10)	**0.002**
Corneal-compensated IOP (Black)	108	Reference		35	-0.16 (-1.85, 1.53)	0.85	12	0.77 (-1.74, 3.28)	0.54
Goldmann-correlated IOP (White)	9 788	Reference		8 708	0.05 (-0.05, 0.15)	0.35	1 684	0.36 (0.18, 0.54)	**<0.001**
Goldmann-correlated IOP (Black)	108	Reference		35	-0.01 (-1.57, 1.55)	0.99	11	-0.47 (-2.85, 1.91)	0.70

* Indicates odds ratio. UKB, UK Biobank; CLSA, Canadian Longitudinal Study on Aging; *N*, sample size; β, beta coefficient; OR, odds ratio; CI, confidence interval.
Small participant numbers and heterogeneity in definitions precluded meaningful analyses of participants classified as 'Other' ethnicity.

Supplementary Table 7. Results of Mendelian randomization analyses for smoking initiation and smoking intensity on corneal hysteresis and corneal resistance factor using all independent genetic variants reported in the original GSCAN publication as instrumental variables

**MR method**	**Corneal hysteresis**		**Corneal resistance factor**	
	Estimate (95% CI)	*P*	Estimate (95% CI)	*P*
Smoking initiation (194 SNPs)				
IVW	0.15 (0.06, 0.24)	**<0.001**	0.08 (-0.02, 0.18)	0.13
Weighted median	0.11 (0.02, 0.20)	**0.014**	0.09 (-0.01, 0.19)	0.07
Weighted mode	0.03 (-0.16, 0.22)	0.10	0.02 (-0.29, 0.32)	0.92
MR-Egger	0.08 (-0.26, 0.43)	0.65	-0.08 (-0.49, 0.33)	0.71
MR-PRESSO	0.13 (0.05, 0.20)	**<0.001**	0.08 (0.00, 0.16)	0.06
Smoking intensity (38 SNPs)				
IVW	0.17 (0.05, 0.28)	**0.005**	0.12 (-0.02, 0.26)	0.09
Weighted median	0.18 (0.03, 0.32)	**0.016**	0.09 (-0.06, 0.25)	0.24
Weighted mode	0.16 (0.02, 0.29)	**0.030**	0.08 (-0.07, 0.22)	0.30
MR-Egger	0.12 (-0.09, 0.32)	0.27	0.08 (-0.17, 0.32)	0.54
MR-PRESSO	–	–	0.10 (-0.03, 0.22)	0.14

MR estimates expressed per unit change in the instrumental variable.
GSCAN, GWAS (genome-wide association study) & Sequencing Consortium of Alcohol and Nicotine use; SNP, single nucleotide polymorphisms; CI, confidence interval; IV, instrumental variable; SNP, single nucleotide polymorphism; IVW, inverse variance weighted; MR, Mendelian randomization; PRESSO, pleiotropy residual sum and outlier.
No MR-PRESSO estimate is calculated if no significant outliers are detected.
